# A study of the benefits and methods of evaluating comprehensive management of residents with premature ventricular contractions in a community hospital

**DOI:** 10.1097/MD.0000000000043252

**Published:** 2025-07-04

**Authors:** Kai Wu, Bangdi Wei, Bin Cai, Jiaqi Liu

**Affiliations:** a Department of Family Medicine, Xidu Community Health Service Center of Fengxian District, Shanghai, China; b Jiangsu Health Vocational College, Nanjing, China; c Shanghai Fengxian District Central Hospital, Shanghai, China; d Hospital 902, Joint Logistics Support Force, Bengbu, China.

**Keywords:** community hospital, Lown score, management model, Myerburg score, premature ventricular contractions

## Abstract

Premature ventricular contractions are the most common ventricular arrhythmias in clinical practice and are most commonly observed in organic heart diseases, such as cardiac insufficiency. Community physicians often lack experience in treating patients with premature ventricular contractions, and there is no standardized method for assessing the risk level of premature ventricular contractions or relevant primary care guidelines to guide community physicians in their practice. We used the premature ventricular contraction score to guide family physicians in the comprehensive management and treatment of community-dwelling patients to find a method of assessing premature ventricular contractions that is suitable for community physicians to operate. Statistical analyses were performed on 350 residents with premature ventricular contractions. The differences in the number of ambulatory electrocardiographic ventricular premature beats at 1, 3, 6, and 12 months of follow-up within each of the control, L low-risk L, L intermediate-risk L, L high-risk L, M low-risk M, M intermediate-risk M, and M high-risk groups were statistically significant (*P* < .05), and the number of ambulatory electrocardiographic ventricular premature beats in each of the groups showed a decreasing trend over the follow-up period. Adverse events occurred 31 times during the follow-up period, and the number of cases of heart failure, ventricular tachycardia, and ventricular fibrillation in group M was significantly higher than that in group L (*P* = .001). The within-group grouping of group L was more sensitive to changes in the number of premature ventricles and discriminated changes in the number of premature ventricles better than group M. The Myerburg score was better than the Lown score for adverse events, and group M was able to better judge the prognosis of premature ventricular failure.

## 1. Introduction

Premature ventricular complexes, also known as premature ventricular beats, are the most common ventricular arrhythmias in clinical practice and are most commonly associated with organic cardiac diseases, such as cardiac insufficiency, but are also not uncommon in the normal population.^[1]^ A Holter monitor found that 39% of normal people had at least 1 premature ventricular beat, and 4% had more than 100 premature ventricular beats in 24 hours.^[2]^ The most common ventricular arrhythmia is premature ventricular beat, which is often associated with cardiac insufficiency. Ventricular prematurity occurs in 39% of normal subjects and in 4% more than 100 times in 24 hours. With increasing age, the prevalence of ventricular prematurity is as high as 69% in people over 75 years of age.^[3]^ In addition to the high correlation between ventricular prematurity and the severity of the underlying disease in patients with organic heart disease, the risk of cardiovascular events and death in patients with frequent ventricular prematurity in the absence of organic heart disease was not significantly different from that in normal subjects in a previous study with a 10-year follow-up; thus, the premature ventricular events in patients with structurally normal heart disease were considered to be benign in the past.^[4–6]^ However, recent studies have found that frequent ventricular prematurity can lead to left ventricular enlargement and dysfunction, and thus, the development of cardiomyopathy and even heart failure.^[7]^ ^[8]^ It has also been shown that premature ventricular contractions observed during the recovery phase of treadmill exercise testing are associated with long-term mortality in patients without structural heart or coronary artery disease.^[9]^ Community physicians are often inexperienced in seeing patients with premature ventricular disease in outpatient clinics, and there are no standardized methods to assess the risk level of premature ventricular disease or related primary care guidelines to guide the diagnostic and therapeutic behaviors of community physicians. Further standardization and investigation are needed to determine when to initiate 2-way referral of patients with premature ventricular disease and the benefits of community-based comprehensive management interventions (regular follow-up, medication guidance, lifestyle interventions, psychological guidance).

## 2. Materials and methods

### 2.1. Study subject and sample selection

In this study, 350 patients with premature ventricular contractions attending the outpatient clinic of our community hospital during the period from June 2020 to June 2023 were taken as the study subjects, and were divided into 300 cases in the intervention group and 50 cases in the control group using simple randomization, and the intervention group was further divided into Group L and Group M based on the Lown score and Myerburg score. There were 75 men and 75 women in group L; age 36 to 91 years old, mean (67.19 ± 10.36) years old; there were 73 men and 77 women in group M; age 43 to 86 years old, mean (65.00 ± 9.42) years old; and there were 27 men and 23 women in the control group; age 44 to 84 years old, mean (65.08 ± 9.14) years old. The inclusion criteria: electrocardiogram was consistent with the diagnosis of premature ventricular contractions^[10]^; life was able to take care of themselves, able to carry out outpatient or telephone follow-up; family members and patients provided informed consent. Exclusion criteria: malignant tumors, serious infections, etc; exclusion of residents with hearing and mental problems; exclusion of residents who have undergone radiofrequency ablation of premature ventricular contractions, implantation of buried cardiac defibrillators, and cardiac synchronization therapy. The general data such as age, sex, and disease duration of the 2 groups of patients were comparable, with no statistically significant difference (*P* > .05). The results are presented in Tables 1 and 2.

**Table 1:** Baseline characteristics of the study participants.

Variables	Control group (n = 50)	L group (n = 150)	M group (n = 150)	*P*
Gender				
Male	27	75	73	.808
Female	23	75	77	
Age, yr	65.08 ± 9.14	67.19 ± 10.36	65.00 ± 9.42	.123
Hypertension	29	116	119	.005
BMI (kg/m^2^)	24.19 ± 2.93	24.57 ± 3.03	24.10 ± 2.97	.371
TG (mmol/L)	1.21 ± 0.54	1.21 ± 0.49	1.26 ± 0.52	.652
TC (mmol/L)	4.11 ± 0.89	4.22 ± 1.08	4.12 ± 1.12	.973
LDL-C (mmol/L)	2.20 ± 0.49	2.63 ± 0.96	2.56 ± 1.00	.060
HDL-C (mmol/L)	1.04 ± 0.26	1.12 ± 0.26	1.18 ± 0.30	.032
FBG (mmol/L)	5.67 ± 0.42	5.74 ± 0.51	5.69 ± 0.49	.522
Currently taking oral medications				
Beta-blocker	24	72	68	.707
Propafenone	14	39	42	
Moresizine	12	39	40	
HAMA	7.48 ± 1.19	10.30 ± 2.70	10.13 ± 2.69	.000

BMI = body mass index, FBG = fasting blood glucose, HAMA = Hamilton Anxiety Scale score, HDL-C = high-density lipoprotein, LDL-C = low-density lipoprotein, TC = cholesterol, TG = triglycerides.

**Table 2:** Comparison of general information in the intervention group.

Variables	L1 (n = 50)	L2 (n = 50)	L3 (n = 50)	M1 (n = 50)	M2 (n = 50)	M3 (n = 50)	*P*
Genders							
Male	25	25	25	23	26	24	.995
Female	25	25	25	27	24	26	
Age, yr	66.54 ± 9.64	65.74 ± 9.65	69.30 ± 11.54	63.92 ± 9.22	65.60 ± 10.62	65.48 ± 8.37	.152
Hypertension	34	37	46	34	39	46	.003
BMI (kg/m^2^)	24.31 ± 3.13	25.18 ± 3.49	24.23 ± 2.32	24.27 ± 3.22	24.28 ± 2.93	23.75 ± 2.77	.311
TG (mmol/L)	1.17 ± 0.48	1.30 ± 0.54	1.16 ± 0.45	1.30 ± 0.52	1.15 ± 0.46	1.34 ± 0.56	.220
TC (mmol/L)	4.02 ± 1.01	4.05 ± 1.03	4.39 ± 1.20	4.18 ± 1.01	4.09 ± 1.18	4.17 ± 1.17	.603
LDL-C (mmol/L)	2.43 ± 0.80	2.48 ± 0.95	2.81 ± 1.14	2.29 ± 0.76	2.58 ± 1.06	2.55 ± 1.12	.293
HDL-C (mmol/L)	1.11 ± 0.24	1.08 ± 0.26	1.20 ± 0.32	1.12 ± 0.32	1.14 ± 0.29	1.22 ± 0.29	.207
FBG (mmol/L)	5.73 ± 0.50	5.80 ± 0.54	5.69 ± 0.49	5.66 ± 0.48	5.67 ± 0.47	5.73 ± 0.51	.745
Currently taking oral medications							
Beta-blocker	26	23	23	25	21	22	.869
Propafenone	13	13	13	14	14	14	
Moresizine	11	14	14	11	15	14	
HAMA	7.10 ± 1.55	8.48 ± 1.17	12.02 ± 2.29	7.66 ± 1.92	8.30 ± 1.19	11.88 ± 2.52	.000

BMI = body mass index, FBG = fasting blood glucose, HAMA = Hamilton Anxiety Scale score, HDL-C = high-density lipoprotein, LDL-C = low-density lipoprotein, TC = cholesterol, TG = triglycerides.

### 2.2. Methods

#### 2.2.1. Clinical data collection

##### 2.2.1.1. Intervention group

300 residents with premature ventricular contractions in the community were selected, and the Lown and Myerburg scores were assessed by electrocardiogram and 24-hour ambulatory electrocardiogram, respectively.^[11,12]^ Lown score grading scale: grade 0, no ventricular presystole; grade I, single episodic ventricular presystole < 30 times/h; grade II: single frequent ventricular presystole ≥ 30 times/h; grade III: multiple origin ventricular presystole; grade IV: paired ventricular presystole or consecutive ventricular presystole ≥ 3; Grade V: RonT ventricular presystole. Grade I: single episodic ventricular presystole < 30 beats/h; Grade II: single frequent ventricular presystole ≥ 30 beats/h; Grade III: multiple ventricular presystoles; Grade IV: paired ventricular presystoles or ≥ 3 consecutive ventricular presystoles; Grade V: RonT ventricular presystole. Myerburg score grading scale: Each subject was scored according to the Myerburg score: no ventricular premature was scored as 1 point in the frequency stratification; rare ventricular premature (<1/h) was scored as 2 points; infrequent ventricular premature (1–9/h) was scored as 3 points; moderate ventricular premature (10–29/h) was scored as 4 points; and frequent ventricular premature (≥30/h) was scored as 5 points. In the morphologic stratification, monomorphism and monogenicity were scored as 1 point; polymorphism and polygenesis were scored as 2 points; repeated paired ventricular tachycardia and short bursts of ventricular tachycardia were scored as 3 points; non-sustained ventricular tachycardia (≥6 beats, duration ≤ 30 s) was scored as 4 points; sustained ventricular tachycardia (duration > 30 s) was scored as 4 points; and the Myerburg score was calculated by adding up the scores of the frequency stratification and the morphologic stratification. Patients with Lown scores of 0 to II were enrolled in the L low-risk group (L1), patients with scores III to IVa in the L intermediate-risk group (L2), and patients with scores IVb to V in the L high-risk group (L3). The Myerburg score A0-B2 was assigned to the M low-risk group (M1), B3-C2 to the M medium-risk group (M2), and C3-E4 to the M high-risk group (M3). For all patients in the intervention group, baseline data on sex, age, body mass index, history of hypertension, fasting glucose, lipids, cardiac ultrasound left ventricular systolic and end-diastolic internal diameters, left atrial internal diameters, left ventricular ejection fraction, current oral medications, and Hamilton Anxiety and Depression Scale assessments were collected. Patients in L1 and M1 were given conventional medication or psychological counseling, and patients with significant anxiety and tension were given anxiolytic and depressive medication applications with regular follow-up. Patients in the L2 and M2 groups were given antiarrhythmic medication, and patients with severe symptoms related to ventricular premature were referred to higher-level hospitals for treatment and prognostic follow-up. Patients in L3 and M3 are promptly referred to higher-level hospitals, the treatment opinions of higher-level hospitals on patients with ventricular premature are recorded, and patients’ survival status and prognosis are regularly followed up.

##### 2.2.1.2. Control group

Patients in the control group were only subjected to the collection of baseline data, such as sex, age, body mass index, history of hypertension, fasting blood glucose, blood lipids, cardiac ultrasound left ventricular systolic and end-diastolic internal diameters, left atrial internal diameters, left ventricular ejection fraction, current oral medications, and assessment of the Hamilton Anxiety and Depression Scale. The control group underwent routine outpatient treatment, and routine management of patients by community physicians was recorded.

##### 2.2.1.3. Methods of follow-up

Both the control and intervention groups underwent outpatient follow-up at 1, 3, 6, and 12 months. Follow-up visits were required to record the patients’ ECG and ambulatory electrocardiograms, their general symptoms, whether they underwent radiofrequency ablation, the occurrence of heart failure, ventricular tachycardia, ventricular fibrillation, implantable cardioverter defibrillator implantation, and adverse events, such as death.

### 2.3. Statistical methods

Count data are expressed as percentages, and comparisons between the 2 groups were made using the chi-square test. Measurement information with normal distribution was expressed as ($\bar{x}\pm s$), independent samples *t*-test was used, and measurement information with non-normal distribution was expressed as [M(Q1, Q3)]. The Mann–Whitney *U* test was used to compare data between the 2 groups. Correlations between variables were analyzed using Spearman rank correlation. All statistical analyses were performed using Statistical Package for SPSS (version 26.0). A *P*-value of <.05 was considered to denote statistical significance..

## 3. Results

### 3.1. Comparison of general information among the 3 groups

Statistical analyses of sex, age, history of hypertension, BMI, TG, TC, LDL-C, fasting glucose, current oral medication, and other relevant indicators among the control group, group L, and group M were not significantly different (*P* > .05). The number of cases with a history of hypertension was significantly higher in groups L and M than in the control group (*P* < .05), the HAMA scores in groups L and M were higher than those in the control group (*P* < .05), and the HDL-C in group M was higher than that in the control group (*P* < .05), which was comparable. The results are presented in Table 1.

### 3.2. Comparison of general information in the intervention group

There was no significant difference in the statistical analysis of sex, age, BMI, TG, TC, LDL-C, HDL-C, fasting blood glucose, current oral medication, and other related indices among the intervention groups such as L1, L2, L3, M1, M2, and M3 (*P* > .05). The number of cases with a history of hypertension was significantly higher in L3 and M3 than in the other groups (*P* < .05). The HAMA scores of L2, L3, M2, and M3 were higher than those of L1, and the HAMA scores of L3 and M3 were higher than those of L2 (*P* < .05). The HAMA scores of L3 were higher than those of M1 and M2 (*P* < .05), and the HAMA scores of M3 were higher than those of M1 (*P* < .05). was higher than that in M2 (*P* < .05). The results are presented in Table 2.

### 3.3. Comparison of echocardiographic indices in 3 groups

From the echocardiographic indices, it was found that the differences in LVEF and LAD among the control, L, and M groups were not statistically significant (*P* > .05). The LVEDD and LVESD of group L and group M were not significantly different (*P* > .05) and were greater than those of the control group (*P* < .05); however, the cardiac ultrasound parameters of all 3 groups were within the normal reference range. The results are shown in Table 3.

**Table 3:** Comparison of echocardiographic indices in 3 groups.

Variables	Control group (n = 50)	L group (n = 150)	M group (n = 150)	*P*
LVEF (%)	65.38 ± 5.45	66.23 ± 5.59	65.6339 ± 5.80	.544
LAD (mm)	37.21 ± 5.77	37.97 ± 5.33	38.39 ± 5.47	.404
LVEDD (mm)	44.60 ± 4.65	46.56 ± 2.79	46.72 ± 2.78	.000
LVESD (mm)	28.86 ± 2.77	31.12 ± 2.84	30.79 ± 3.70	.000

LAD = left atrial internal diameter, LVEDD = left ventricular end-diastolic internal diameter, LVEF = left ventricular ejection fraction, LVESD = left ventricular end-systolic internal diameter.

### 3.4. Comparison of echocardiographic indices in the intervention group

The echocardiographic indices showed that the differences in LVEF and LAD among the intervention groups were not statistically significant in the control, L1, L2, L3, M1, M2, and M3 groups (*P* > .05). LVEDD was significantly higher in L3 and M3 than in the control, L1, and M1 groups (*P* < .05), and LVEDD was significantly higher in M3 than in L2 and M2 groups (*P* < .05). LVESD was significantly higher in L1, L2, L3, M2, and M3 than that in the control group (*P* < .05), and LVESD was significantly higher in L3 and M3 than that in L1, M1, M1, and M2 (*P* < .05). The results are presented in Table 4.

**Table 4:** Comparison of echocardiographic indices in the intervention group.

Variables	Control group (n = 50)	L1 (n = 50)	L2 (n = 50)	L3 (n = 50)	M1 (n = 50)	M2 (n = 50)	M3 (n = 50)	*P*
LVEF (%)	65.38 ± 5.45	65.35 ± 5.62	67.31 ± 5.29	66.02 ± 5.78	65.20 ± 5.72	65.63 ± 5.99	66.07 ± 5.76	.491
LAD (mm)	37.21 ± 5.77	38.65 ± 5.04	37.36 ± 5.46	37.88 ± 5.52	37.77 ± 5.09	38.48 ± 5.70	38.92 ± 5.64	.692
LVEDD (mm)	44.60 ± 4.65	45.65 ± 2.96	46.62 ± 2.54	47.41 ± 2.55	45.60 ± 2.62	46.36 ± 2.73	48.21 ± 2.30	.000
LVESD (mm)	28.86 ± 2.77	30.05 ± 2.72	30.94 ± 2.35	32.35 ± 2.92	28.95 ± 3.73	30.05 ± 3.09	33.36 ± 2.64	.000

LAD = left atrial internal diameter, LVEDD = left ventricular end-diastolic internal diameter, LVEF = left ventricular ejection fraction, LVESD = left ventricular end-systolic internal diameter.

### 3.5. Changes in symptoms of premature beats before and after 12 months of treatment

Prior to treatment, there was no statistically significant difference between the groups in terms of premature beat symptom profiles within each of the control, L1, L2, L3, M1, M2, and M3 groups (*P* = .149). After 12 months of follow-up, the symptomatic conditions of premature beats within each group of the control group, L1, L2, L3, M1, M2, and M3, were significantly reduced compared to the pretreatment period, with a significant difference (*P* < .05). The results are presented in Table 5.

**Table 5:** Changes in symptoms of premature beats before and after 12 mo of treatment.

Groups	Quantities	Treatment node	Symptomatic	Asymptomatic	*P*
Control group	50	Before treatment After 12 mo	44 26	6 24	.000
L1	50	Before treatment After 12 mo	41 29	9 21	.000
L2	50	Before treatment After 12 mo	44 27	6 23	.000
L3	50	Before treatment After 12 mo	48 23	2 27	.000
M1	50	Before treatment After 12 mo	40 28	10 22	.000
M2	50	Before treatment After 12 mo	44 25	6 25	.000
M3	50	Before treatment After 12 mo	47 22	3 28	.000
*P*					.149*

* *P* is the case of whether there is a difference in the comparison of the number of cases in each group before treatment.

### 3.6. Changes in the number of premature ventricular beats at 24 hours

As shown in Tables 6 and 7, before treatment, the difference in the number of premature ventricular beats on ambulatory electrocardiograms among the control, L1, L2, L3, M1, M2, and M3 groups was not statistically significant. After the above stages of treatment, the differences in the number of ambulatory electrocardiographic premature ventricular beats between and within the control, L1, L2, L3, M1, M2, and M3 groups were statistically significant at the 1-, 3-, 6-, and 12-months posttreatment follow-ups.

**Table 6:** Variation in the number of premature ventricular beats in 24 h (comparison within groups).

Variables	Control group (n = 50)	L1 (n = 50)	L2 (n = 50)	L3 (n = 50)	M1 (n = 50)	M2 (n = 50)	M3 (n = 50)
Before treatment	12,693.64 ± 1752.00	13,070.83 ± 1818.11	12,984.99 ± 1913.07	12,568.14 ± 1919.36	12,566.70 ± 1821.43	12,689.29 ± 1899.20	12,806.78 ± 1844.02
1 mo	10,982.92 ± 1414.47*	11,033.35 ± 1749.57*	10,520.87 ± 1930.41*	11,389.73 ± 1815.23*	10,517.30 ± 1640.71*	10,733.93 ± 1600.24*	10,720.03 ± 1755.43*
3 mo	8085.20 ± 938.77*†	7937.55 ± 1096.11*†	7909.35 ± 1060.94*†	8128.07 ± 1113.82*†	7989.40 ± 915.31*†	8044.47 ± 1001.87*†	8131.71 ± 1029.29*†
6 mo	5891.18 ± 512.24*†‡	4410.08 ± 431.35*†‡	4805.93 ± 515.78*†‡	5374.73 ± 457.66*†‡	4907.85 ± 430.85*†‡	4936.07 ± 438.81*†‡	5088.40 ± 419.20*†‡
12 mo	3832.66 ± 288.28*†‡§	2467.11 ± 247.48*†‡§	2824.97 ± 274.73*†‡§	3299.98 ± 226.59*†‡§	3215.20 ± 261.95*†‡§	3265.26 ± 216.14*†‡§	3303.73 ± 235.88*†‡§
*P*	.000	.000	.000	.000	.000	.000	.000

* *P* < .01 compared with pretreatment.

† *P* < 0.01 compared with 1 mo.

‡ *P* < 0.01 compared with 3 mo.

§ *P* < 0.01 compared with 6 mo.

**Table 7:** Variation in the number of premature ventricular beats in 24 h (comparison between groups).

Variables	Control group (n = 50)	L1 (n = 50)	L2 (n = 50)	L3 (n = 50)	M1 (n = 50)	M2 (n = 50)	M3 (n = 50)	*P*
Before treatment	12,693.64 ± 1752.00	13,070.83 ± 1818.11	12,984.99 ± 1913.07	12,568.14 ± 1919.36	12,566.70 ± 1821.43	12,689.29 ± 1899.20	12,806.78 ± 1844.02	.759
1 mo	10,982.92 ± 1414.47	11,033.35 ± 1749.57	10,520.87 ± 1930.41	11,389.73 ± 1815.23	10,517.30 ± 1640.71	10,733.93 ± 1600.24	10,720.03 ± 1755.43	.131
3 mo	8085.20 ± 938.77	7937.55 ± 1096.11	7909.35 ± 1060.94	8128.07 ± 1113.82	7989.40 ± 915.31	8044.47 ± 1001.87	8131.71 ± 1029.29	.896
6 mo	5891.18 ± 512.24	4410.08 ± 431.35*	4805.93 ± 515.78*†	5374.73 ± 457.66*†‡	4907.85 ± 430.85*‡§	4936.07 ± 438.81*‡§	5088.40 ± 419.20*†‡§	.000
12 mo	3832.66 ± 288.28	2467.11 ± 247.48*	2824.97 ± 274.73*†‡	3299.98 ± 226.59*†‡	3215.20 ± 261.95*†‡	3265.26 ± 216.14*†‡	3303.73 ± 235.88*†‡	.000

* *P* < .01 compared with control group.

† *P* < .01 compared with L low-risk group.

‡ *P* < .01 compared with L intermediate-risk group.

§ *P* < .01 compared with L high-risk group.

The difference in the number between the control, L, and M groups was not statistically significant (*P* > .05) after 1 and 3 months of follow-up, but the difference in the number between the control, L, and M groups was statistically significant (*P* < .01) after 6 and 12 months of follow-up.

After 6 months of follow-up, the number of times in the control group was higher than that in the L and M groups (*P* < .01), and within-group analysis showed that the number of times in L1 was lower than that in L2 and L3 (*P* < .01), and the number of times in L2 was lower than that in L3 (*P* < .01), whereas the difference in the number of times within the groups in the M group was not statistically significant (*P* > .05).

After 12 months of follow-up, the number of times in the control group was higher than that in both the L and M groups (*P* < .01), and within-group analysis showed that the number of times in L1 was lower than that in both L2 and L3 (*P* < .01), and the number of times in L2 was lower than that in L3 (*P* < .01), while the difference in the number of times within the various groups of the M group was not statistically significant (*P* > .05).

The differences in the number of ambulatory electrocardiographic ventricular premature beats at 1, 3, 6, and 12 months of follow-up within each group of the control, L1, L2, L3, M1, M2, and M3 groups were statistically significant (*P* < .05), and the number of ambulatory electrocardiographic ventricular premature beats in each group showed a decreasing trend over the follow-up period.

### 3.7. Comparison of adverse events

31 adverse events occurred during the follow-up period, of which one case of heart failure occurred in the control group. The number of heart failure, ventricular tachycardia, ventricular fibrillation, ICD implantation, and death in groups L and M were 1, 2, 1, 0, and 10, 11, 5, and 0, respectively, and the number of heart failure, ventricular tachycardia, and ventricular fibrillation in group M was significantly higher than that in group L (*P* = .001). The results are presented in Table 8.

**Table 8:** Comparison of adverse event rates.

Groups	Adverse events				*p*
	HF	VT, VF	ICD	Deaths	.001
Control group	1	0	0	0	
L group	1	2	1	0	
L group	10	11	5	0	

HF = heart failure, VF = ventricular fibrillation, VT = ventricular tachycardia.

## 4. Discussion

Premature ventricular contractions (hereinafter referred to as premature ventricular contractions) are a common clinical arrhythmia, which refers to the presence of ectopic pacing points located in the ventricle below the bifurcation of the Hirschsprung bundle. The issuance of abnormal, premature electrical impulses, resulting in early heart depolarization, early beating phenomenon, especially in coronary artery disease, cardiomyopathy, hypertensive heart disease, rheumatic heart disease, and patients with organic heart disease, such as mitral valve prolapse, are more common, and the main mechanisms are categorized as autoregulatory abnormalities, triggered activity, and refractoriness. The disease is characterized by episodes accompanied by symptoms such as panic, chest tightness, dizziness, and fatigue, while ECG shows repeated changes with a high degree of variability. In clinical practice, it is not difficult to diagnose the disease using ECG examination and symptom presentation. However, the treatment and prognosis of premature ventricular disease are difficult to assess because of the difficulty in accurately identifying the specific pathogenesis of the disease in individuals.

In patients with organic heart disease, ventricular premature is highly correlated with the severity of the underlying disease. In addition, in a previous study of patients with frequent ventricular premature in the absence of organic heart disease followed up for 10 years, the risk of cardiovascular events and death was not significantly different from that of normal people, so the past viewpoint is that premature ventricular occurs in patients with normal cardiac structure. However, recent studies have found that frequent ventricular prematurity can lead to left ventricular enlargement and dysfunction, and thus, the development of cardiomyopathy and even heart failure.^[13,14]^ In more serious cases, premature ventricular death can impact the stability of the patient’s blood circulation and even lead to sudden death. According to statistical data, the incidence of malignant ventricular arrhythmia is as high as 62% to 80% in patients with sudden cardiac death,^[15,16]^ and as age increases, the incidence of premature ventricular arrhythmia is as high as 69% in people over 75 years of.^[13,17]^

For a long time, China’s community health service centers have undertaken more chronic disease management, maternal and child management, vaccination, and other services. With the accelerating aging of the population, the demand for cardiovascular disease diagnosis and treatment services is rising rapidly, and primary healthcare institutions are expected to become the “main battlefield” for the diagnosis and treatment of chronic cardiovascular diseases. However, in outpatient clinics of community health centers, community doctors are relatively inexperienced in treating patients with premature ventricular disease. The risk assessment methods for patients with premature ventricular disease and the relevant primary care guidelines are not standardized enough to guide the diagnostic and therapeutic behaviors of community doctors. Therefore, it is important to know when to make 2-way referrals for patients with VT and when to make comprehensive management interventions in the community (e.g., regular follow-up, medication guidance, lifestyle interventions, and psychological counseling). In the present study, 2 different rating methods, the Lown score and Myerburg score, were used to group the patients with premature ventricular disease, and the general data and adverse events were statistically analyzed to assess the advantages and disadvantages of the 2 scoring methods, and to evaluate which method is more suitable for comprehensive management interventions in community hospitals.

In a comparison of the general data, the number of cases with a history of hypertension was significantly higher in the Lown group (hereinafter referred to as L group) and Myerburg group (hereinafter referred to as M group) than in the control group; the number of cases with a history of hypertension was higher in the L3 and M3 groups than in the other groups, which suggests that hypertension is a risk factor for premature ventricular disease, and that the degree of risk of premature ventricular disease is prone to be higher in patients with a combined history of hypertensive disease. Studies have shown that long-term hypertension increases the load burden on the left ventricle, decreases ventricular compliance, and results in changes in the geometric configuration of the left ventricle, which in turn causes cardiomyocyte hypertrophy and focal interstitial fibrosis, and ultimately, an increase in ventricular wall thickness and stiffness inevitably occurs, and most ventricular arrhythmias have a direct relationship with myocardial hypertrophy and interstitial fibrosis.^[18–20]^ At the cellular level, essential hypertension leads to electrical and structural remodeling of the ventricles, impaired gap junctions between cells, resulting in abnormal cellular signaling and unstable electrical activity of the heart, and concomitant changes in the formation of pathological substrates such as ventricular hypertrophy, fibrosis, and scarring. Alterations in these mechanisms induce ventricular arrhythmias.^[21–23]^ The results of 1 study showed that patients with LV hypertrophy had significantly higher scores on the Lown classification than those without LV hypertrophy, suggesting that the presence of LV hypertrophy and myocardial ischemia in patients with essential hypertension can lead to an increase in the incidence of premature ventricular arrhythmias,^[24]^ which is in agreement with our results and further confirms the role of the mechanism of hypertensive disorders in the development of premature ventricular arrhythmias.

Our study showed that Hamilton Anxiety Scale (HAMA) scores were higher in the L and M groups than in the control group, and HAMA scores were higher in L3 and M3 than in the other groups, suggesting that the higher the score, the higher the risk of ventricular premature birth, and the more likely that the state of anxiety predisposition will exist. Relevant studies have found that negative emotions such as anxiety and depression can cause plant dysfunction, stimulate the hypothalamic-pituitary-adrenal system, activate the downstream sympathetic nerves, and increase the release and activity of catecholamines, leading to an increase in the abnormal arrhythmia of cardiomyocytes that are originally not arrhythmic, and at the same time, sympathetic excitation can also lead to the reduction of perceptual domains and the reduction of heart rate variability, which ultimately induces the occurrence of various cardiac arrhythmias.^[25]^ The results of a retrospective study that included 1514 patients with social phobia and 4542 control patients showed that patients with social phobia were at a higher risk of arrhythmia after covariate adjustment, and the rate of arrhythmia detection was higher in patients with anxiety or depression than in patients without abnormal mentalities.^[26,27]^ Carney et al demonstrated that heart rate variability is reduced after myocardial infarction in patients with depression. Increased sympathetic tone following reduced HRV decreases the threshold for ventricular fibrillation.^[28]^ In addition, severe myocardial ischemia and necrosis caused by acute myocardial infarction (AMI) will cause extreme sympathetic hyperactivity and reduced vagal excitability. Negative emotions such as anxiety and depression further activate sympathetic excitability on this basis, resulting in a significant decrease in vagal activity and severe autonomic dysfunction. Severe dysfunction of the autonomic nervous system can cause disturbances in cardiac electrical activity, which can lead to ventricular tachycardia, ventricular fibrillation and ventricular tachycardia.^[28]^ Ventricular tachycardia, ventricular fibrillation, and even sudden cardiac death with AMI.^[29–32]^ These studies have demonstrated that patients with social phobia or suffering from anxiety and depression have a higher risk of arrhythmias and are more likely to develop different types of arrhythmias. In addition, the symptoms of ventricular tachycardia vary widely in clinical practice, with the majority of patients having no obvious symptoms; however, occasional ventricular tachycardia can cause serious symptoms, including palpitations, chest tightness, and a feeling of cardiac arrest. Partial ventricular prematurity can lead to decreased cardiac output and insufficient perfusion to vital organs, which may cause fatigue, shortness of breath, sweating, dizziness, blackouts, and even angina attacks, while rhythm disturbances caused by ventricular capture may easily lead to heart palpitations. Ventricular tachycardia is often characterized by symptoms of chest tightness and palpitations and can recur without any obvious triggers. Since many patients still do not have enough knowledge about ventricular tachycardia and are more sensitive to the onset of symptoms, a considerable number of patients are prone to anxiety and depression, and the dysfunction of autonomic regulation of the heart is more serious in patients with arrhythmia accompanied by anxiety,^[33,34]^ which in turn aggravates ventricular tachycardia episodes and symptoms and negatively affects the effectiveness of the treatment to a certain extent.

In addition to the conventional application of antiarrhythmic drugs, we also administered anxiolytic and depressive drugs according to the results of the HAMA score combined with the presence or absence of obvious symptoms of tension and anxiety. At the 1-, 3-, 6 months and 12 months follow-up, the differences in the number of ventricular premature beats on ambulatory electrocardiograms within the control group, L1, L2, L3, M1, M2, and M3 were statistically significant, and the number of ventricular premature beats on ambulatory electrocardiograms in each group showed a decreasing trend over the follow-up time. In addition, after 12 months of follow-up, the number of premature ventricular beats in each group was significantly reduced compared with the pretreatment period, indicating that individualized drug treatment (combination of various antiarrhythmic and anxiolytic drugs) and timely referral can significantly reduce the number of premature ventricular beats and improve the clinical symptoms of the patients. Currently, many drugs are commonly used in the treatment of premature ventricular disease, including propafenone, moracizine, and β-blockers, which are widely used in the clinic and have good therapeutic effects. Clinically, anxiety and depression are common emotional responses that are prevalent in cardiovascular diseases.^[35–37]^ Therefore, in addition to the application of appropriate antiarrhythmic drugs, it is necessary to correctly assess patients’ anxiety and depression, perform HAMA scoring, and intervene according to the scoring and actual situation of patients. Changes in 5-hydroxytryptamine levels in the body can cause changes in the sensitivity of the corresponding receptors, which in turn can lead to changes in microvascular diastolic function of the heart. Since structural abnormalities are delayed, changes in the neuroendocrine system often precede changes in cardiac structure, and some studies have found that patients with cardiac neurosis are at risk of developing cardiac insufficiency, myocardial ischemia, or cardiac arrhythmias.^[38–40]^ Currently, selective serotonin reuptake inhibitors (SSRIs) with safety evidence are recommended as first-line antidepressants and anxiety medications in several studies and guidelines and are currently recommended as first-line medications for patients with coronary atherosclerotic heart disease because of their mild anticholinergic and cardiac side effects. medication. A JAMA REMIT study found that SSRIs reduced the incidence of mental stress induced myocardial ischemia (MSIMI) in patients with coronary artery disease and MSIMI. By setting up a double-blind controlled experiment, this study found that the incidence of MSIMI in the experimental group was significantly lower than that in the placebo group, and that the use of SSRIs not only changed the psychological state of patients but also improved the physiological function of the heart.^[41]^ In addition, compared with traditional antidepressants (e.g., tricyclic antidepressants), SSRIs are better tolerated and have essentially the same level of efficacy. For patients with coronary artery disease, evidence suggests that sertraline, citalopram, and fluoxetine are effective in improving depressive symptoms with fewer side effects. For patients with heart failure, sertraline, escitalopram, citalopram, and paroxetine have all been clinically studied.^[42,43]^ Sertraline has been found to significantly reduce the number of ventricular premature beats, enhance patients’ quality of life, and heart rate variability in nondepressed patients with ischemic heart failure and implantable cardioverter-defibrillators.^[44]^ In addition, γ-aminobutanoic acid (GABA) plays an important role in the human cerebral cortex, hippocampus, thalamus, basal ganglia, and cerebellum and has a regulatory effect on a variety of functions of the organism; when there is a lack of GABA in the human body, it produces anxiety, restlessness, fatigue, apprehension, and other emotions. Numerous animal studies have shown that spinal cord stimulation therapy can inhibit sympathetic signals induced by myocardial ischemia and stabilize the outflow of cardiac efferent signals, thereby reducing ventricular arrhythmias during ischemia.^[45]^ By establishing a model of acute ischemia in Yorkshire pigs, Kimberly et al electrically stimulated the thoracic spinal cord of the model thereby activating the GABA signaling pathway and reducing ischemia-reperfusion-induced sympathoexcitation and arrhythmias, and additionally Kimberly et al found that GABA transaminase inhibitors, when given intrathecally alone, provided a cardioprotection similar to that of spinal cord stimulation therapy,^[46]^ with GABA aminotransferase inhibitors increased synaptic GABA concentrations by inhibiting GABA aminotransferase and provided protection against ventricular arrhythmias after myocardial infarction by activating GABA-B receptors.^[47]^ The results of 1 study showed that, after the use of anxiolytic and depressive therapy in patients with ventricular preterm contraction, although the degree of ventricular preterm contraction did not significantly improve, somatic symptoms such as chest tightness and palpitations significantly improved, and the number of preterm contraction episodes was significantly reduced.^[48]^

Clinical studies have shown that excessive premature ventricular load (>10 000 beats/24 h or > 10% of the total cardiac beats) may cause cardiac enlargement and reduced ejection fraction.^[49]^ Therefore, the reduction of premature ventricular load using methods such as pharmacologic or catheter ablation is of great clinical significance. In this study, the left ventricular end-diastolic diameter (LVEDD) was significantly higher in L3 and M3 than in the control group, L1, and M1. The left ventricular end-systolic diameter (LVESD) was significantly higher in L3 and M3. diameter (LVESD) was significantly higher in L3 and M3 than in the control, L1, L2, M1, and M2; however, it was within the normal range. It has been found that frequent ventricular prematurity lead to an increase in ineffective myocardial work production, causing insidious mechanical bradyarrhythmias, compensatory enlargement of the left ventricle, and reduced systolic function,^[1,50]^ suggesting that high loads of ventricular prematurity are far more detrimental to the heart’s structure and function than low or intermediate loads. On the other hand, our study suggests that the higher the score, the higher the premature ventricular load. The ventricular premature load was found to be a predictor of the development of ventricular premature-induced cardiomyopathy. Ultrasound speckle tracking imaging revealed that patients with a ventricular premature load of 8% to 10% began to show mild impairment of myocardial function, a ventricular premature load of 10% resulted in reversible cardiomyopathy, a ventricular premature load of 24% was the most valuable cutoff value for distinguishing between ventricular premature-induced or non-ventricular premature-induced cardiomyopathy, and a ventricular premature load of 24% was independently associated with ventricular premature-induced cardiomyopathy. Premature ventricular-induced cardiomyopathy is independently associated.^[51,52]^

In this study, after 6 months of follow-up, the number of ventricular preterm episodes in the control group was higher than that in both the L and M groups within-group analyses showed that the number of ventricular preterm episodes in L1 was lower than that in both L2 and L3, the number of ventricular preterm episodes in L2 was lower than that in L3, and the difference between the number of ventricular preterm episodes in each of the groups within the M group was not statistically significant. In contrast, after 12 months of follow-up, the number of premature ventricles in the control group was higher than that in groups L and M. Within-group analyses showed that the number of premature ventricles in L1 was lower than that in both L2 and L3, the number of premature ventricles in L2 was lower than that in L3, and the difference between the number of premature ventricles within each group of group M was not statistically significant. In addition, after 12 months of follow-up, the symptomatic condition of premature contractions within each group was significantly reduced compared to that before treatment, indicating that the number of premature ventricles can be significantly reduced through appropriate clinical diagnosis and treatment plan, individualized medication (combined with the application of anti-anxiety medication), and timely referral measures. It also suggests that by grouping patients with low, medium, and high risks of premature ventricular disease by Lown score and Myerburg score, medium- and high-risk patients can be effectively identified and treated according to their actual conditions. This suggests that by grouping, the identification of premature ventricles by both methods is better than that of the control group, and the intragroup grouping of group L is more sensitive to the change in the number of premature ventricles, while the difference in the number of premature ventricles within each group of group M was not statistically significant, so that the discrimination of the change in the number of premature ventricles in group L was better than that in group M.

Risk classification of premature ventricular disease is a method that helps predict the prognosis of patients and formulate treatment plans. At present, there are various scoring methods, such as the Lown, Myerburg, Schamaroth, and Hoffmayer integral methods. At this stage, the Lown and Myerburg score methods are more commonly used in clinical as well as scientific research.^[53,54]^ In 1971, Lown and Wolf studied the electrocardiographic monitoring data of 220 patients who suffered from AMI during hospitalization and proposed the Lown classification scheme, which classifies ventricular premature by marking the duration and number of premature ventricular episodes. This is a simple, easy-to-use method that has been widely used in clinical practice. However, it did not take into account the morphological characteristics of premature ventricles and overemphasized the risk of R-on-T, and its results were prone to bias.^[55]^ In 1984, Myerburg et al proposed the Myerburg classification scheme to assess premature ventricular contractions. The Myerburg classification is a method that classifies the frequency and morphology of ventricular arrhythmias in parallel, and provides a more comprehensive prognosis by qualitative and quantitative analysis of the correlation between qualitative and quantitative indexes and the patient’s prognosis. It is a better classification method for patients with chronic heart disease score method for the risk of premature ventricular disease.^[56]^ The Premature Ventricular Contraction Scoring System significantly improves diagnostic and treatment efficiency as well as patient safety by scientifically stratifying complex clinical information into actionable prognostic indicators. The Lown score, for example, can help distinguish high-risk patients who need intervention from benign ventricular premature beats that do not require treatment, and frequent ventricular premature beats (≥30 beats/hour or > 2000 beats in 24 hours) is an independent risk factor for poor prognosis, as well as identifying high-risk ventricular premature beats that are prone to induce ventricular tachycardia and ventricular fibrillation. There are existing studies on the use of the temporal and amplitude characteristics of P-, QRS-, and T-waves to predict obstructive coronary artery disease using machine learning. Future studies should incorporate artificial intelligence and big data analysis to develop more accurate multidimensional scoring models to address the need for individualized medicine.^[57,58]^ This study showed that among the adverse events that occurred during the follow-up period, the number of cases of heart failure, ventricular tachycardia, and ventricular fibrillation in group M was significantly higher than that in group L, which indicated that the Myerburg score was better than the Lown score in judging the adverse events, and it could better judge the prognosis of ventricular premature.

## 5. Conclusion

To summarize, the higher the risk level of ventricular premature birth, the more likely it is to be combined with hypertension disease, and the more likely they are to have a state of anxiety, it is important to pay attention to the control of hypertension in clinical practice. The higher the score of ventricular premature birth, the higher the load of ventricular premature birth, and individualized medication and timely referral measures can significantly reduce the number of ventricular premature births and improve the clinical symptoms of patients. In addition, the tendency of the 2 different methods to assess ventricular premature is different, and each has its own advantages and disadvantages. The within-group grouping of group L was more sensitive to changes in the number of premature ventricles and discriminated changes in the number of premature ventricles better than group M. The Myerburg score was better than the Lown score for adverse events, and group M was able to better judge the prognosis of premature ventricular failure.

## Acknowledgments

We would like to thank all the general practitioners working in community health service centers in Shanghai for the online questionnaire interviews conducted in this study.

## Author contributions

**Data curation:** Kai Wu, Bangdi Wei.

**Investigation:** Bin Cai.

**Project administration:** Bin Cai, Jiaqi Liu.

**Resources:** Jiaqi Liu.

**Software:** Kai Wu.

**Supervision:** Jiaqi Liu.

**Writing – original draft:** Kai Wu, Bangdi Wei.

**Writing – review & editing:** Jiaqi Liu.
